# Spinal corollary discharge modulates motion sensing during vertebrate locomotion

**DOI:** 10.1038/ncomms8982

**Published:** 2015-09-04

**Authors:** Boris P. Chagnaud, Roberto Banchi, John Simmers, Hans Straka

**Affiliations:** 1 Department of Biology II, Ludwig-Maximilians-University Munich, Grosshadernerstrasse 2, 82152 Planegg-Martinsried, Germany; 2 Graduate School of Systemic Neurosciences, Ludwig-Maximilians-University Munich, Planegg-Martinsried 82152, Germany; 3 Institut de Neurosciences Cognitives et Intégratives d'Aquitaine, Université de Bordeaux, CNRS UMR 5287, 33076 Bordeaux, France

## Abstract

During active movements, neural replicas of the underlying motor commands may assist in adapting motion-detecting sensory systems to an animal's own behaviour. The transmission of such motor efference copies to the mechanosensory periphery offers a potential predictive substrate for diminishing sensory responsiveness to self-motion during vertebrate locomotion. Here, using semi-isolated *in vitro* preparations of larval *Xenopus*, we demonstrate that shared efferent neural pathways to hair cells of vestibular endorgans and lateral line neuromasts express cyclic impulse bursts during swimming that are directly driven by spinal locomotor circuitry. Despite common efferent innervation and discharge patterns, afferent signal encoding at the two mechanosensory peripheries is influenced differentially by efference copy signals, reflecting the different organization of body/water motion-detecting processes in the vestibular and lateral line systems. The resultant overall gain reduction in sensory signal encoding in both cases, which likely prevents overstimulation, constitutes an adjustment to increased stimulus magnitudes during locomotion.

## Abstract (web summary)

Corollary discharges inform the central nervous system about impending motor activity. Here, Chagnaud *et al*. show that, in *Xenopus* tadpoles, shared efferent neural pathways to the inner ear and lateral line adjust the sensitivity of sensory afferents during locomotor activity.

## Graphical abstract

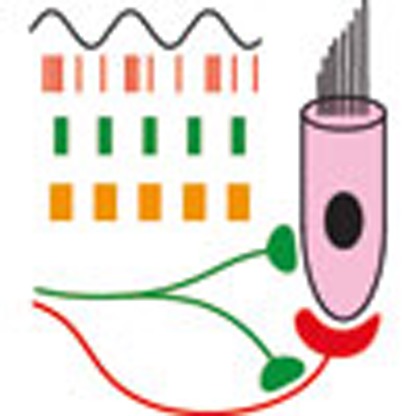

The efficient encoding of sensory information requires matching neural coding processes to ongoing variations in stimulus parameters^1^,^2^,^3^. A particularly relevant example of a sensory system that experiences a wide range of constantly altering stimulus amplitudes and frequencies is the vestibular system, the major sensor of body motion in vertebrates^4^. Vestibular endorgans decompose an animal's movement into spatio-temporal vector components as a prerequisite for correct visual orientation, postural control and spatial navigation^5^. Accordingly, the accurate sensing of body motion-derived stimuli and their processing within the central nervous system depends critically on neuronal computations that ensure the optimal encoding of static and changing head/body positions in space^6^. Locomotor activity thus poses a particular challenge for the vestibular system, given the necessity to detect and encode a wide dynamic range of body motion to which the coding process must adapt.

A convenient way for mechanosensory encoding to be adaptively adjusted during self-motion is through the employment of corollary discharge or efference copies originating from the locomotor neural centres themselves. In contrast to reactive sensory-derived processes, the predictive nature of these intrinsic feed-forward signals is well suited to inform associated sensory systems at various levels of the nervous system about impending and/or ongoing motor activity^7^,^8^,^9^,^10^. In this context, vertebrates possess a highly suitable neuronal substrate for a peripheral gain control mechanism that can tune hair cell sensitivity and adapt afferent encoding in the movement-detecting periphery of both the vestibular and lateral line sensory systems^11^,^12^. Populations of hindbrain efferent neurons (ENs) innervate the hair cells and primary afferent fibres of vestibular endorgans, and exclusively the hair cells of lateral line neuromasts^11^,^13^,^14^,^15^. Moreover, for lateral line neuromasts—the sensors of water displacement in fish and aquatic amphibians—the efferent innervation is already known to affect afferent neuron discharge during locomotion-related behaviour^16^,^17^,^18^,^19^, although the origin and precise nature of the transmitted efferent signal thus far remain unknown.

Although various aspects of vestibular efferent pathway activity and its influence on inner ear endorgan receptors have been described in both anamniote^20^,^21^,^22^,^23^,^24^ and amniote vertebrates^25^,^26^,^27^,^28^,^29^, the findings have been divergent and even contradictory when compared across different experimental approaches or species. For instance, the effect of direct vestibular efferent activation on vestibular afferents has been reported to be exclusively excitatory in monkeys and fish, both excitatory and inhibitory in frogs^30^, and at variance with the consistently reported inhibitory efferent influence on afferent fibre discharge in the lateral line system^18^,^19^. Furthermore, although vestibular efferents have been proposed to convey signals related to anticipated head/body motion, ongoing sensory stimulation^31^,^32^ or arousal states before feeding, attack or escape^21^,^31^, there has hitherto been only sparse indications of a causal relationship between vestibular efferent firing patterns and altered afferent signalling during an identified natural behaviour.

One of the best examples of vestibular efferent influence on sensory encoding is the increase in vestibular afferent discharge found in toadfish in response to touch, light or sound stimulation^21^. However, the spinalization of experimental animals in this study prevented any association between vestibular efferent activity and a spinal-driven motor behaviour. Moreover, during active head motion in primates, there is no evidence for any vestibular efferent-mediated effect on the afferent responses to movement, thereby further leaving the question of a spinal motor circuit influence on peripheral sensing unanswered^28^. Thus, despite some evidence for a behavioural context-specific role, a general functional picture of these efferent systems and their impact on mechano-afferent encoding has so far remained elusive.

Since locomotion constitutes a major source of stimulation for the vestibular system, neural activity related to the generation of locomotor output is potentially well suited to access and modulate the vestibular periphery via the latter's efferent innervation. Here, we provide direct evidence in larval *Xenopus* frogs that ascending corollary discharge signals originating from central pattern generator (CPG) circuitry in the spinal cord are conveyed to mechanosensory ENs of both the vestibular and lateral line systems during rhythmic locomotor activity. The phase-coupled discharge of these efferent pathways transmits the temporal structure of the locomotor CPG output and causes an overall gain reduction in afferent encoding of concomitant sensory inputs to both systems.

## Results

### ENs are rhythmically active during locomotion

**Figure 1:**
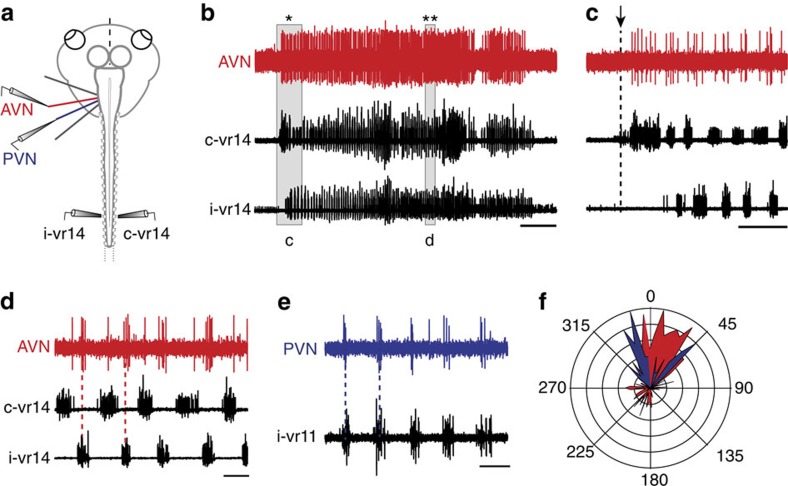
Locomotor-related neural activity in vestibular nerve efferent neurons in *Xenopus* tadpoles. (**a**–**d**) Episodes of spontaneous fictive swimming in semi-isolated *in vitro* preparations (**a**), recorded as multiple-unit impulse discharge (**b**–**d**) in the left (ipsilateral) and right (contralateral) ventral roots (i-vr and c-vr, respectively; black traces) of spinal segment 14 together with the central cut portion of the left anterior vestibular (VIII^th^) nerve branch (AVN, red trace). The initial discharge at episode onset (*) and subsequent regular (**) vr bursting (shaded areas in **b**) are shown on an extended timescale in **c** and **d**, respectively. After mostly tonic firing at swim episode onset (**c**), the AVN activity develops into rhythmic bursting occurring in phase with locomotor bursts in the ipsilateral vr (red dashed lines in **d**). (**e**) Different preparation showing coincident burst coupling between ipsilateral vr11 and the posterior vestibular nerve (PVN) branch (blue dashed lines) during an episode of fictive swimming. (**f**) Polar plot quantifying the phase relationship between the i-vr/AVN and i-vr/PVN activity shown in **d** and **e**; AVN (red area) and PVN bursts (blue area) are approximately in phase (angle towards 0°) with the i-vr burst rhythm. Calibration bars: 5 s in **b**, 1 s in **c**, 0.2 s in **d** and **e**.

The neural correlate of undulatory tail-based swimming in *Xenopus* tadpoles is expressed as spontaneous, left/right-alternating impulse bursts in spinal ventral roots (vrs) of semi-isolated *in vitro* preparations (Fig. 1a–e). Such episodes of so-called ‘fictive locomotion' typically consist of an initial irregular discharge at episode onset (black traces in Fig. 1b,c) followed by a more regular, bilaterally symmetrical vr burst rhythmicity (Fig. 1b,d) that persists for up to tens of seconds at a frequency of 2–8 Hz).

Single- and multiunit recordings of the central severed ends of the anterior (AVN) or posterior branch (PVN) of the vestibular (VIIIth cranial) nerve (Fig. 1a) revealed the occurrence of locomotor activity-timed discharge in both of these otherwise silent mechanosensory nerves (Fig. 1b–e; Supplementary Fig. 1b,c). Following a short tonic firing at swim episode onset (red traces in Fig. 1b,c), the two vestibular nerve branches displayed sustained rhythmic discharge that was closely timed with spinal vr motor bursting on the same side of the cord (dashed vertical lines in Fig. 1d,e; Supplementary Fig. 1b,c). The strict in-phase coordination of AVN and PVN discharge with ipsilateral vr burst activity and their out-of-phase relationship with contralateral vr bursts was confirmed by circular plot analysis of instantaneous vr firing relative to spiking in both vestibular nerves recorded on the same side (PVN, blue and AVN, red in Fig. 1f; Supplementary Fig. 1d,e). It is noteworthy, however, that in many preparations the predominant ipsilateral coupling between spinal vr and vestibular/lateral line nerve activity could be transiently replaced by a biphasic pattern where mechanosensory nerve discharge occurred in phase with the rhythmic vr bursts on both cord sides (see AVN recording in Fig. 1d and Supplementary Fig. 1b).

An identical coupling relationship with spinal vr bursting was also observed for the anterior (ALLN) and posterior nerves (PLLNs) of the neighbouring lateral line system during fictive locomotion (Supplementary Fig. 1f–j), consistent with earlier reports on the activation of lateral line efferent fibres during swimming in both *Xenopus* and dogfish^16^,^17^,^19^. Significantly, however, the coupling of lateral line (as well as vestibular) nerve activity with spinal vr bursts observed in our motionless semi-isolated preparations extends on these previous studies by excluding sensory feedback signals as a potential source of the rhythmic efferent signal during locomotion. Moreover, this common locomotor influence provided us with the unique opportunity to explore in parallel and directly compare the efferent control of the two co-existing mechanosensory systems under the same experimental conditions within the same animal.

Although mechanosensory afferent axons considerably outnumber the relatively small efferent fibre population in the vestibular and lateral line nerves^15^, the rhythmic bursting observed in these cranial nerves during fictive swimming could be directly established to reflect a central activation of individual ENs by locomotor-related signals. Support for this conclusion derived from simultaneous recordings of the central and peripheral regions of the vestibular and lateral line nerves after an intervening transection close to their exit from the brainstem (for example, see configuration for PLLN recordings in Supplementary Fig. 2a). Axons in the central stump displayed rhythmic burst discharge in phase with fictive swimming (blue trace in Supplementary Fig. 2b) but otherwise remained silent at rest or during hydrodynamic stimulation of the skin (red trace in Supplementary Fig. 2b). In contrast, spontaneous firing occurred in the detached distal nerve segment at rest as well as during fictive locomotion, but was strongly sensitive to mechanical skin stimulation (green trace in Supplementary Fig. 2b). These separate discharges recorded peripherally and centrally from vestibular and lateral line nerves thus corresponded to the dissociated activities of mechanoreceptor afferent and efferent axons, respectively.

**Figure 2:**
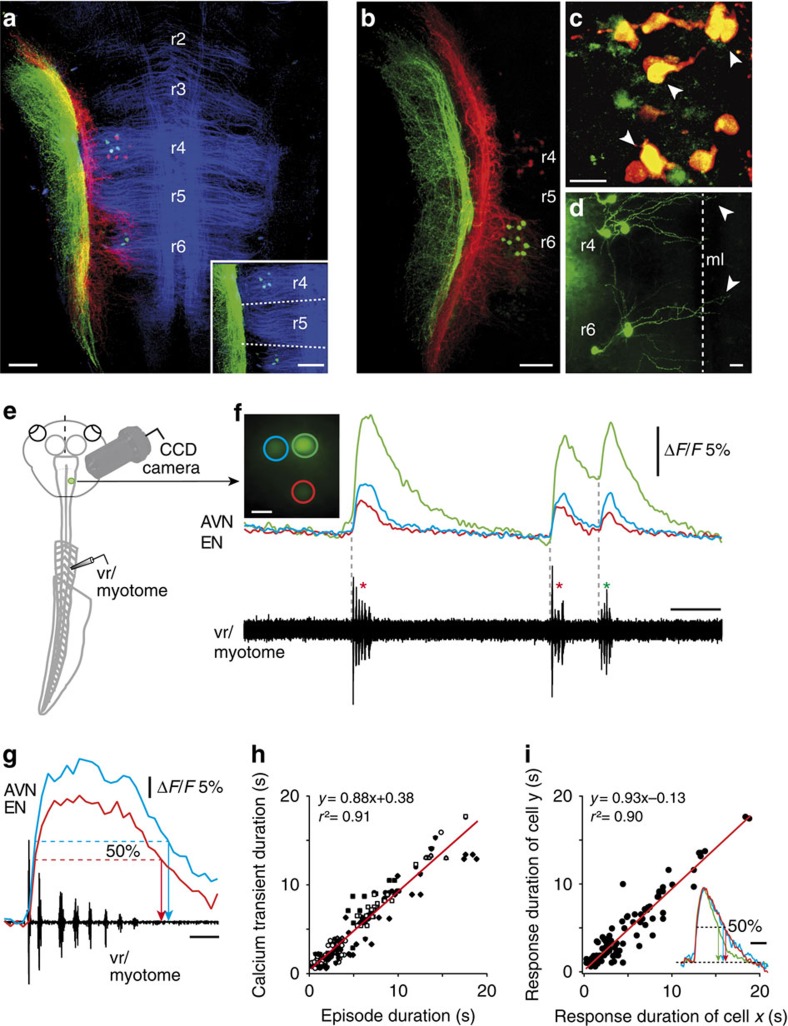
Ca^2+^ imaging of morphologically identified mechanosensory efferent neurons (ENs) during fictive locomotion. (**a**–**d**) Confocal reconstructions of hindbrain whole mounts after combined application of Alexa Fluor 546 (red) and 488 (green) dextran to the AVN and ALLN (**a**,**c**,**d**) and to the AVN and PLLN, respectively (**b**) showing afferent axonal projections and locations of EN somata in rhombomeres (r) 4 and 6. Note that the longitudinal and rhombomere-specific transverse fibres (blue in **a**) were visualized by 612 nm illumination. The inset in **a** shows r4 and r6 ALLN ENs (green) in relation to segmental boundaries (dashed lines). (**c**) AVN (red) and ALLN (green) ENs in r4 at higher magnification; note the double-labelled neurons in yellow (arrowheads). (**d**) ALLN ENs in r4 and r6 that extend dendrites (arrowheads) across the midline (ml). (**e**,**f**) Imaging of Ca^2+^ transients in ENs of semi-isolated preparations (**e**) following retrograde loading of cell bodies with Calcium Green-1 dextran from the anterior vestibular nerve (AVN). Ca^2+^ transients (**f**) were recorded simultaneously in several ENs (colour-coded cells and traces) during episodes of evoked (red *) and spontaneous (green *) ventral root/myotomal locomotor burst activity (black traces). (**g**,**h**) Correlation between Ca^2+^ dynamics, measured as the overall response half-width (**g**) and corresponding locomotor episode duration (**h**) in 32 cells during 4–7 swimming episodes per monitored cell (*n*=160). (**i**) Plot of Ca^2+^-response durations (measured as half-width; see colour-coded, normalized transients in inset) of 25 EN pairs during 4–7 locomotor episodes per cell (*n*=114). Red lines in **h** and **i** represent linear regression. Calibration bars: 0.1 mm in **a** and **b** and inset in **a**; 25 μm in **c** and **d**; 15 μm in **f**; 5 s for traces in **f** and inset in **i**; 0.2 s in **g**.

In a next step, the central nervous location and relative positions of vestibular and lateral line ENs was determined by retrograde double labelling with fluorescent tracers (Alexa Fluor 488 and 546 dextran) applied to different combinations of the two VIIIth nerve branches and the two lateral line nerves in individual preparations (Fig. 2a–d). Whereas vestibular and lateral line afferent axons terminated separately in adjacent areas of the hindbrain (red- and green-labelled fibres, respectively, in Fig. 2a,b), the somata of conjointly labelled ENs formed overlapping subgroups of 5–12 cells per preparation (AVN: 7.8±4.6, *n*=20; PVN: 7.7±1.5, *n*=3; ALLN: 7.8±2.8, *n*=10; PLLN: 9.2±3.4, *n*=6) aligned ipsilaterally in rhombomeres (r) 4 and 6 (Fig. 2a–d). The dendritic tree of this bipartite cell population extended predominantly into the ipsilateral reticular formation, although a few branches were found to cross the midline (arrowheads in Fig. 2d). While ENs with axonal projections in the two vestibular nerve branches and the PLLN were confined to single segments (r4 and r6, respectively), ALLN efferents form a larger subgroup (∼70%) in r4 and a smaller population in r6, wherein they intermingled with PLLN EN somata. Moreover, the majority (∼80%) of the ENs in r4 expressed double labelling (see arrowheads in Fig. 2c) after combined tracer application to different combinations of the AVN, PVN and ALLN. The extent of this double labelling was unrelated to mechanosensory nerve branch identity, complying with a previous proposal that individual ENs project to multiple hair cell targets^15^,^33^.

To determine the proportion of neurons within the combined lateral line and vestibular efferent population that become activated during locomotion, we used Ca^2+^ imaging to monitor intrasomatic Ca^2+^ fluctuations associated with electrophysiologically recorded fictive swimming (Fig. 2e). EN cell bodies in r4 were retrogradely loaded with a Ca^2+^ sensor (Calcium Green-1 dextran) from the AVN (Fig. 2f; see Methods). During episodes of both evoked and spontaneous fictive swimming (red and green * in Fig. 2f), all backfilled cells (32 ENs in 7 preparations) exhibited coincident fluorescence changes with onsets that were strictly timed to the onset of rhythmic spinal vr bursting (Fig. 2f,g). The duration of these responses, measured as the half-width of the overall Ca^2+^ signal (Fig. 2g), was also closely correlated with the duration of the corresponding fictive swimming episode (Fig. 2h). Moreover, the dynamics of the Ca^2+^ responses of different EN pairs (*n*=20 from a total of 25 cells) during a given episode were very similar and highly correlated (Fig. 2i), suggesting a common underlying synaptic drive. Given the projection of individual ENs to multiple peripheral targets, and the close similarities of their Ca^2+^ transients (Fig. 2f,i) and firing patterns during rhythmic vr bursting (Fig. 1d,e; Supplementary fig. 1b,c,i,j), it is probable that the entire efferent population participates in conveying a copy of spinal CPG activity to the inner ear and lateral line sensory peripheries during swimming.

### Information content of the locomotor signal in ENs

**Figure 3:**
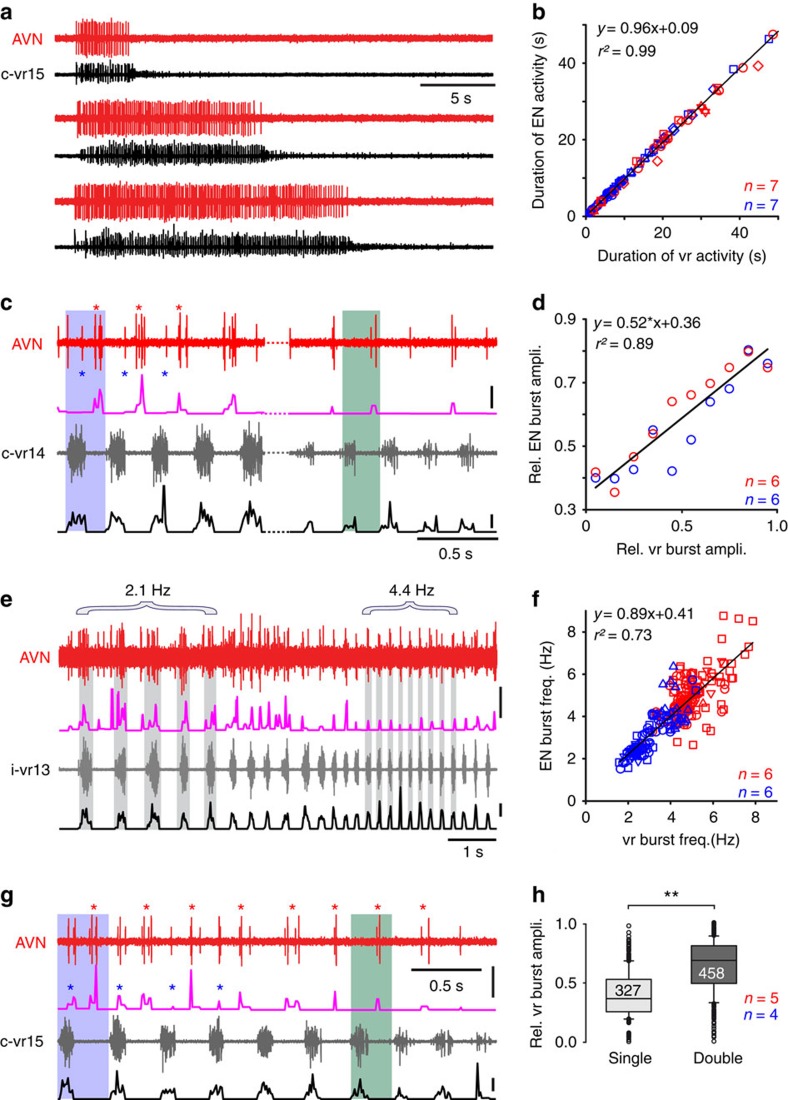
Parameter representation of locomotor activity in EN discharge. (**a**) Recordings of AVN (red) and c-vr15 activity (black) during swimming episodes of increasing length in the same preparation. (**b**) Pooled data plot showing matching episode durations of vestibular (red, *n*=7 preparations) and lateral line (blue, *n*=7 preparations) EN versus vr discharge (68 episodes; black line: linear regression). (**c**) Recordings of AVN (red) and c-vr14 activity (grey) with corresponding integrals of intra-burst firing rates (pink and black traces) during sequences of strong (left) and weak fictive swimming (right) within the same episode (compare colour-shaded areas). During strong swimming (left), additional spikes occurred in the AVN (blue *) in phase with the contralateral vr. (**d**) Group data plot showing a close correlation between the magnitudes of vestibular (red circles, *n*=6 preparations) and lateral line (blue circles, *n*=6) EN versus vr burst integrals (313 burst cycles; black line: linear regression,). (**e**) Recording of AVN (red trace) and i-vr13 activity (grey trace) with respective integrals of intra-burst firing rates (pink and black traces) during a swim episode where vr bursting changed spontaneously from a slower (2.1 Hz) to a faster (4.4 Hz) rhythm. (**f**) Group plot showing a close correlation between vestibular (red, *n*=6) and lateral line (blue, *n*=6) EN versus vr burst frequencies (101 cycles of 5–10 bursts per episode). Note that any biphasic EN burst patterns were omitted from this analysis (black line: linear regression). (**g**) Recording of AVN (red) and c-vr15 activity (grey) with corresponding firing rate integrals (pink and black) during a swimming episode in which the single-phase vr-EN coupling (red * in **g**) followed a pattern of EN activity occurring in time with the vr bursts on both sides (red and blue * in **g**). (**h**) Box and whisker plots showing that the biphasic EN activity (blue) occurred with vr bursts of significantly larger relative magnitude (**P≤0.001; Mann–Whitney *U*-test) than during monophasic EN-vr coupling. Number (*n*) of preparations is indicated in **b**,**d**,**f** and **h**. Vertical calibration bars in **c**,**e** and **g** indicate a discharge rate of 100 spikes per second.

The corollary discharge activation of mechanosensory ENs offers the possibility of transmitting information about a range of different features of the propulsive motor commands to the vestibular and lateral line sensory peripheries. Moreover, given the communal projections of most ENs to both systems, it is predictable that equivalent efferent information is conveyed in the nerve branches to the two peripheral targets. As typified by the AVN recordings in Fig. 3a, spinal vr and vestibular (Fig. 3b, red plots) or lateral line EN activity (Fig. 3b, blue plots) during fictive swimming revealed a close temporal match (*r*^2^=0.99) in their overall discharge durations in each episode (Fig. 3a,b; see also Fig. 2h). Alterations in the strength of actual swimming *in vivo* derive from changes in the amplitude and frequency of horizontal tail excursions that in turn are represented *in vitro* by variations in the discharge intensity and cycle frequency of underlying vr bursts^34^. Spontaneous changes in vr intra-burst firing rates (see shaded c-vr14 bursts in Fig. 3c) and burst frequency (see i-vr13 bursts in Fig. 3e) during fictive swimming were also accompanied by similarly graded alterations in burst magnitude and cycle rate of associated vestibular EN activity (AVN in Fig. 3c,e, respectively). The strong linear correlations for both discharge intensity (Fig. 3d) and burst frequency (Fig. 3f)—obtained from vestibular (red plots) and lateral line (blue plots) nerve recordings—indicate that swimming strength is faithfully represented on a cycle-to-cycle basis within the efferent activity to the vestibular and lateral line peripheries.

Changes in swimming strength are also inscribed in a further parameter of the corollary discharge of mechanosensory ENs. During vr bursting at lower cycle frequencies and/or lower amplitudes (green highlighted areas in Fig. 3c,g), vr/EN coupling consisted predominantly of an ipsilateral, single-phase pattern. However, when vr burst frequency or amplitude (blue shadings in Fig. 3c,g) was relatively high, the coupling pattern was typically a biphasic relationship in which the ENs were now activated along with vr bursts on both cord sides (red and blue * in Fig. 3c,g). Concomitant with this biphasic pattern, the ipsilateral vr/EN coupling became stronger and more pronounced and was correlated with an increase in the relative magnitude of vr burst amplitudes (*P*≤0.01, Mann–Whitney *U*-test; Fig. 3h; single phase: *n*=327 and biphasic: *n*=458). Thus, during stronger swimming, the corollary signature expressed by mechanosensory ENs during each cycle derived from a combination of ascending signals that were in phase with vr burst activity on both sides of the spinal cord. However, even when biphasic coupling occurred during a given locomotor episode, the dominant ipsilateral phase relationship between vr and EN burst discharge was strictly maintained, and independent of rhythm frequency. Together, therefore, the above findings show that during tadpole swimming, the efference copy encoded in both vestibular as well as lateral line ENs conveys information about the duration, frequency and amplitude of locomotor activity to the mechanosensory periphery of the inner ear and lateral line systems.

### Origin of locomotor corollary discharge in ENs

**Figure 4:**
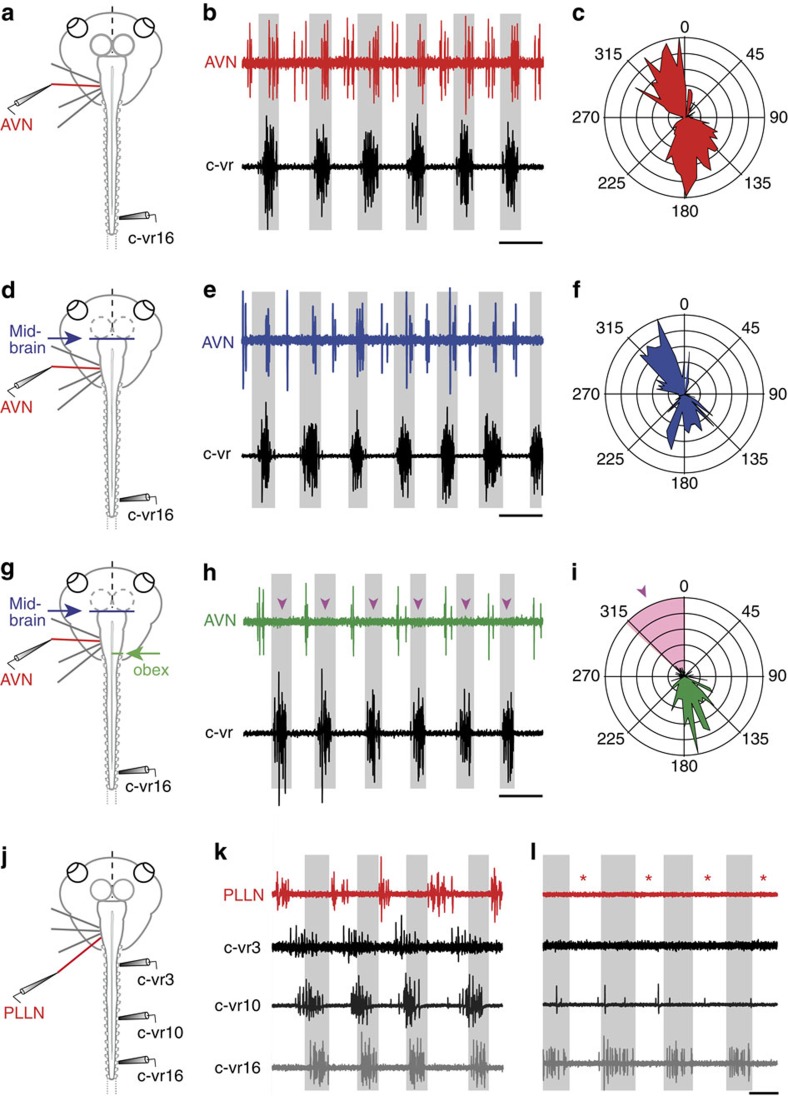
Spinal origin and trajectory of ascending pathways mediating locomotor corollary discharge signalling to ENs. (**a**–**i**) Episodes of spontaneous fictive swimming in the same *in vitro* preparation (**a**,**d**,**g**) recorded from the right (contralateral, c) vr16 (black trace) and the central stump of the left AVN (**b**,**e** and **h**: red, blue and green traces, respectively) in control (**a**–**c**) after midbrain removal (**d**–**f**) and then after a right obex hemisection (**g**–**i**) The corresponding polar plots in **c**, **f** and **i** show that the out-of-phase (contralaterally timed) vr-EN coupling remained largely unaffected by the two lesions. However, although the additional synchronous (ipsilaterally timed) EN activity (**b**,**c**) persisted after midbrain removal (**e**,**f**), it disappeared after obex hemisection (pink arrowheads in **h** and pink area/arrowhead in **i**). (**j**–**l**) Episodes of spontaneous swimming activity in a different semi-isolated preparation (**j**) recorded simultaneously from right vrs 3, 10 and 16 (respectively, black, dark-grey and light-grey traces) and the central stump of the left PLLN (red trace). PLLN bursting coupled with locomotor bursts in all three cord segments (**k**) disappeared (**l**; red *) when CPG burst activity ceased spontaneously in vr3 and vr10, but persisted in vr16. Calibration bars: 0.2 s in **b**,**e**,**h**,**k** and **l**.

In theory, the locomotor-timed influence on mechanosensory ENs, as illustrated in Fig. 4a–c, could originate from the spinal CPG circuitry itself or from supraspinal levels, such as midbrain^35^,^36^ or hindbrain reticular centres^17^ known to control locomotor behaviour. A midbrain contribution was excluded by surgical removal of the midbrain in isolated brainstem/spinal cord preparations (blue arrow in Fig. 4d; *n*=7). Despite the midbrain ablation, the rhythmic activation of both vestibular as well as lateral line ENs persisted during fictive swimming (for example, AVN in Fig. 4e). Moreover, neither the magnitude of locomotor-related EN firing nor the biphasic relationship with left/right vr bursting was affected by this lesion (Fig. 4e,f; *cf.* 4b,c), compatible with a spinal origin of the corollary signal, at least under our *in vitro* experimental conditions. Significantly, however, following an additional spinal cord hemisection at the level of the obex (Fig. 4g), any biphasic EN firing (Fig. 4b,e) was immediately replaced by a single-phase pattern (*n*=10) in which EN/vr coupling remained uniquely ipsilateral as indicated in Fig. 4h,l (*cf.* Fig. 4e,f) by the remaining EN activity occurring in phase-opposition with the contralateral vr. The suppression of EN activation in phase with contralateral vr bursts by this hemisection (see arrowheads and the pink-shaded segment in Fig. 4h,i; *cf.* Fig. 4f) thus suggests that ascending spinal signals reach contralateral ENs after traversing the midline above the obex in the brainstem, thus excluding a previously suggested contribution of the hindbrain reticular formation^16^,^17^. Interestingly, a potential anatomical substrate for this contralateral input could include the midline-crossing dendrites of the mechanosensory ENs themselves (see Fig. 2d).

The origin and coupling dynamics between spinal CPG circuitry and the ENs were further assessed by recording spinal vr activity at different segmental levels during spontaneous episodes of *in vitro* fictive swimming (*n*=5). Robust phase-coupled cyclic bursts occurred in efferent fibres (for example, PLLN in Fig. 4j,k) whenever rhythmic locomotor activity was uniformly expressed along the cord (see vr3, vr10 and vr16 in Fig. 4j). However, locomotor corollary firing in ENs disappeared in all preparations (red * in Fig. 4l; *n*=5) whenever bursting in the most rostral vrs (segments 1–10) occasionally ceased (vr3 and vr10 in Fig. 4l), although bursting in more caudal roots persisted (vr16 in Fig. 4l). Moreover, in preparations expressing typical axially distributed CPG activity (as in Fig. 4k), the stepwise surgical removal of spinal segments, starting at the level of vr20 and continuing rostrally up to vr5, resulted in a gradual reduction of EN burst magnitudes during fictive swimming. Together these findings thus confirm for the first time that the locomotor efference copy drive to both lateral line and vestibular ENs principally derives from CPG circuitry in the rostral cord region.

### Impact of locomotor corollary discharge on sensory encoding

**Figure 5:**
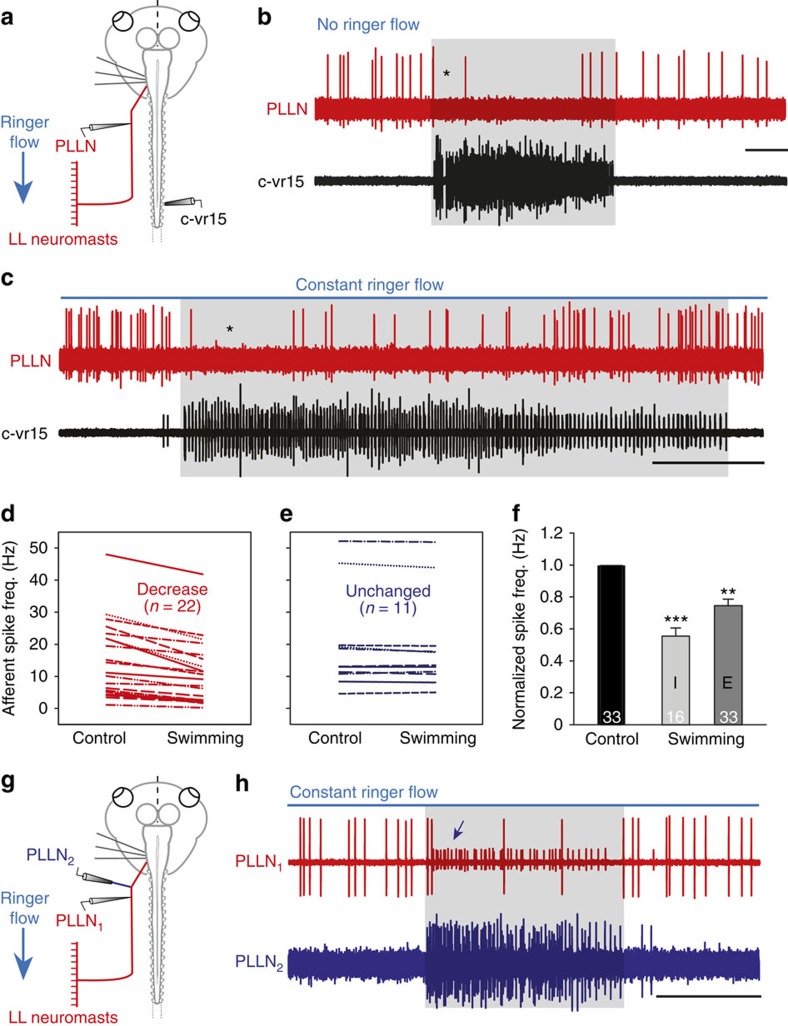
Locomotor corollary discharge influence on lateral line sensory encoding. (**a**–**c**) Simultaneous recordings of right vr15 (black traces) and *en passant* recordings of afferent fibres in the left PLLN (red traces) of a semi-isolated preparation with intact neuromast–lateral line nerve connectivity (**a**) in the absence (**b**) and presence (**c**) of constant sensory stimulation by Ringer flow along the skin surface. During episodes of fictive swimming (shaded areas in **b** and **c**; see black vr trace), tonic PLLN activity was initially abolished (* in **b** and **c**), but regained lower levels of firing as the locomotor activity progressed and eventually returned to resting discharge levels before episode termination. (**d**,**e**) Plots illustrating a decrease (**d**, red) or absence of locomotor influence (**e**, blue) on the firing of individual lateral line afferents during CPG activity. (**f**) Histograms (error bars represent s.e.m.) showing significant average decreases in firing rates of the recorded afferent population (number indicated in each bar) during the initial phase (I) and throughout the entire (E) duration of fictive swimming episodes. **P≤0.001; ***P≤0.0001 (Wilcoxon signed-rank test). (**g**,**h**) Distinguishable afferent and efferent fibre activity in a single PLLN (**g**) recorded *en passant* from one branch (PLLN_1_, red trace) connected to lateral line hair cells and from a second transected branch (PLLN_2_, blue trace) of the same nerve (**h**). During locomotor-related efferent activity (shaded area; indicated by the multiple-unit discharge in PLLN_2_ and spiking in the single small unit (arrow) in PLLN_1_), ongoing afferent firing (large spikes in PLLN_1_) was virtually suppressed throughout the swim episode. Horizontal calibration bars: 5 s in **b** and **c** and 1 s in **h**.

**Figure 6:**
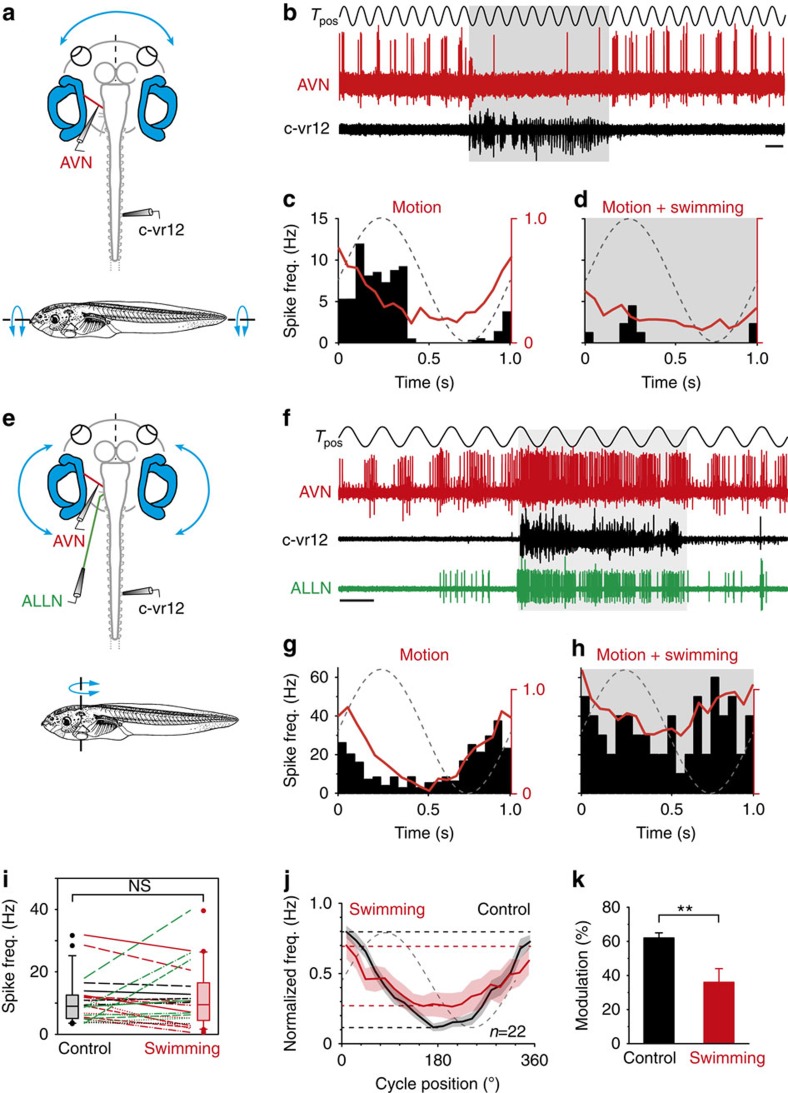
Locomotor corollary discharge influence on vestibular sensory encoding. (**a**–**h**) Recordings of right vr12 (black traces in **b** and **f**), the left ALLN (green trace in **f**) and *en passant* recordings of afferent fibres in the left AVN (red traces in **b** and **f**) in semi-isolated preparations with intact inner ear hair cell–vestibular nerve connectivity (**a**,**e**) during sinusoidal (1 Hz, ±60° s^−1^) horizontal-axis roll motion (**a**) or vertical-axis head rotations (**e**) imposed by a two-axis turntable (*T*_pos_). During fictive swimming (shaded areas in **b** and **f**), the firing of some vestibular afferent fibres was attenuated (**b**–**d**), but facilitated in others (**f**–**h**). (**c**,**d**,**g**,**h**) Histogram (black bars) depicting the mean afferent firing rate modulation (responses of fibres recorded in **b** and **f**) over a single cycle of turntable motion (dashed lines) in the absence (**c**,**g**) and presence (**d**,**h**) of locomotor CPG activity; also plotted are the respective population averages (red curves) of fibres with decreasing (**c**,**d**) and increasing firing (**g**,**h**) during swimming activity. (**i**) Plots of individual mean firing rate alterations during motion stimulation (green—increase, red—decrease and black—no change) before (control) and during swimming activity. Grey and red boxes with whisker plots show the distributions of the average firing rates in the two conditions. NS, not significant. (**j**,**k**) Averaged response of all recorded afferent fibres (±s.e.m., shaded areas in **j**; *n*=22) over a single cycle of turntable motion (dashed grey line) before (black plot) and during (red plot) locomotor CPG activity (**j**). (**k**) Averaged extent (error bars represent s.e.m.) of firing rate modulation before (control) and during fictive swimming. (**k**) **P≤0.001 (Wilcoxon signed-rank test). Horizontal calibration bar in **b** and **f**: 1 s.

The functional consequences of EN locomotor efference copy for sensory signal processing by lateral line neuromasts and vestibular endorgans were explored by making *en passant* recordings from afferent fibres in the PLLN and AVN. For this, the peripheral connectivity of ENs with lateral line/inner ear hair cells and their afferent innervation were left physically intact (Figs 5a,g and 6a,e), in contrast to the experimental conditions described so far (Figs 1, 2, 3, 4) where the endorgans were disconnected. Consequently, the effect of spinal CPG corollary discharge on the transduction and encoding of motion-driven afferent activity during fictive locomotion could be directly assessed. It is also noteworthy that while the application of rotational or hydrodynamic stimuli to the employed semi-isolated preparations did not perfectly mimic natural *in vivo* conditions, it nonetheless allowed evaluating the influence of efferent system activation *in vitro* on the afferent encoding of imposed head/body or water motion, respectively.

### Lateral line system

Previous *in vivo* studies on adult *Xenopus*^17^ and dogfish^16^,^19^ provided qualitative evidence for an attenuating role of EN activity on lateral line afferent signal encoding^16^,^17^,^19^. To extend these earlier observations and directly compare the consequences of locomotor efference copies on vestibular and lateral line primary afferent signalling, we first quantified the EN influence in the latter mechanosensory system. In the absence of locomotor activity, intact PLLN afferent fibres fired spontaneously at overall rates varying from 3 to 50 Hz, depending on the number of afferents recorded in a given experiment (red traces in Fig. 5b,c). During a bout of fictive swimming (see black vr trace in Fig. 5b,c), the discharge of most recorded lateral line afferent neurons (*n*=22/33) became substantially reduced or even ceased completely (* in Fig. 5b,c). The afferent firing rate decrease (Fig. 5d), which was most pronounced immediately after the onset of swimming (see bar I in Fig. 5f) when rhythmic vr bursting was typically at its strongest, generally persisted for most of the ensuing episode (see histogram E in Fig. 5f). A return to control discharge levels often occurred as vr burst amplitudes gradually declined towards episode termination (see Fig. 5c). In contrast to their dependence on the strength of swimming activity (as reflected in the intensity of vr bursting), afferent fibre firing rates were similarly reduced in the absence (Fig. 5b) or presence (Fig. 5c) of a concomitant flow of Ringer solution across the skin surface. While a smaller group of lateral line afferent recordings (*n*=11/33) exhibited no or minimal (<10%) change in spontaneous discharge during fictive swimming (Fig. 5e), an actual increase in afferent cell firing during rhythmic vr bursting was never encountered, consistent with the results of previous studies in which lateral line efferent axons were stimulated electrically^19^. Consequently, the average firing rate of the entire recorded population of lateral line afferents (*n*=33) displayed a significant reduction (*P*≤0.001; Wilcoxon signed-rank test) throughout episodes of locomotor CPG activity (bar E in Fig. 5f).

Simultaneous recordings of lateral line afferent and efferent fibres with intact central and peripheral synaptic connectivity further substantiated the suppressive influence of EN locomotor corollary discharge on mechanosensory afferent neuron firing (Fig. 5g). Very occasionally in such experiments (*n*=3), it was possible to record the activity of pairs of afferent and efferent axons in the same lateral line nerve. In the very rare example shown in Fig. 5h, simultaneous recordings were made from two branches of the same PLLN; an afferent and efferent fibre were recorded *en passant* in the still intact branch (red trace, PLLN_1_ in Fig. 5h) and several ENs alone were recorded with a different electrode placed on a second, severed branch of the same PLLN (blue trace, PLLN_2_ in Fig. 5h). During an episode of EN activity, visible as a barrage of rhythmic discharge in PLLN_2_ (blue trace in Fig. 5h), the firing of an individual lateral line afferent fibre recorded in PLLN_1_ (large spikes in the PLLN_1_ trace in Fig. 5h) was reversibly suppressed. The close temporal correlation between this suppression of afferent firing and the efferent corollary volley (*cf*. PLLN_1_ and PLLN_2_ traces in Fig. 5h) was especially evident from the activity profile of a single efferent fibre (small spikes in the PLLN_1_ trace in Fig. 5h; blue arrow) that accompanied the afferent axon in the *en passant* electrode recording. These reciprocating firing patterns of afferent and efferent axons within the same PLLN branch therefore further support the conclusion that locomotor corollary discharge in mechanosensory ENs is responsible for attenuating sensory signalling in lateral line afferent pathways.

### Vestibular system

The shared projections of individual ENs to both the lateral line and vestibular sensory periphery (see Fig. 2c) also strongly suggested an action of locomotor efference copy on the encoding of motion-related signals in vestibular nerve afferent fibres. This possibility was tested by mounting semi-isolated preparations with still functional vestibular endorgans on a two-axis turntable for the application of rotational stimuli in different spatial planes^37^ (Fig. 6a,e). *En passant* recorded afferent fibres in the AVN fired spontaneously at rest with rates of 220 Hz in different experiments (Supplementary Fig. 3a,c,e). Imposed sinusoidal vertical roll motion (upper trace in Fig. 6b) or horizontal left–right oscillations (upper trace in Fig. 6f) caused a corresponding cyclic modulation of the discharge in all recorded AVN afferent fibres. The peak firing rates of individual recordings during the application of rotational stimuli ranged from 10 to 35 Hz (Fig. 6c,g).

During an episode of fictive swimming in the absence of motion stimulation, the spontaneous firing of vestibular afferents was variably affected, with the discharge rates of some fibres increasing while in others spiking decreased relative to resting levels (see red traces in Supplementary Figs 3a,b and 3c,d, respectively). Consequently, when averaged over all recordings, neither the frequency (*P*=0.067; Wilcoxon signed-rank test; *n*=6) nor the regularity (cv^2^; *P*=0.57; Wilcoxon signed-rank test; *n*=6) of spontaneous discharge was significantly altered during locomotor activity (S′ in Supplementary Fig. 3e,f) compared with the respective controls (C and C′ in Supplementary Fig. 3e,f).

Compatible with a variable impact of spinal CPG corollary activity on afferent fibre resting discharge, a similarly disparate influence of locomotor activity was observed in response to coincident, motion-induced vestibular activation. During a bout of fictive swimming, evidenced by an episode of rhythmic vr bursting (black traces in Fig. 6b,f) and/or associated corollary activity in mechanosensory ENs (ALLN, green trace in Fig. 6f), the ongoing modulation of afferent fibre discharge by sinusoidal rotational stimulation was affected differently in different recordings (shaded areas in Fig. 6b,f). As a first estimate, we calculated the mean firing rate during table motion in the absence and presence of a fictive swimming event; the mean afferent firing rate (red line in Fig. 6c,d,g,h) throughout a given swim episode either decreased (*n*=10; Fig. 6c,d), increased (*n*=7; Fig. 6g,h) or remained unaffected (change<10%; *n*=5). Again, because of this variable influence of locomotor activity during rotational stimulation (colour-coded plots in Fig. 6i), the mean firing rates of the overall afferent population (black and red box plots in Fig. 6i) during (11.0±1.6 Hz; *n*=22) and in the absence of fictive swimming (11.8±2.1 Hz; *n*=22) were not significantly different (*P*=0.434; Wilcoxon signed-rank test). However, irrespective of the diverse spinal CPG influences on individual afferent fibres, any firing rate alteration was always strictly associated with corollary activation of lateral line and vestibular ENs (see the ALLN green trace in Fig. 6f, for example), further pointing to the causality between vestibular/lateral line efferent firing and changes in mechanosensory afferent encoding.

Assuming representivity of the sampled vestibular afferent population, the overall alteration in stimulus-induced discharge modulation observed in our experiments provided a reasonable estimate of the global impact of locomotor corollary discharge on vestibular system movement encoding (Fig. 6j). This became particularly obvious from calculating the average peak-to-peak discharge modulation for a single motion cycle (Fig. 6c,d,g,h,j). Significantly, although mean firing rate levels were variably affected in different fibres by locomotor corollary activity (Fig. 6i), the peak-to-peak amplitudes of the motion-induced modulation were consistently diminished in all recordings compared with controls (compare red with black lines in Fig. 6j). Indeed, the average magnitude of discharge modulation during swimming was significantly reduced by ∼45% (*P*≤0.05; Wilcoxon signed-rank test; *n*=22) with respect to controls (Fig. 6j,k), thereby revealing a considerable reduction in the gain of afferent fibre sensory responsiveness during spinal CPG activity. Thus, together with a comparable impact on the lateral line system, this finding leads to the conclusion that locomotor corollary discharge conveyed by efferent pathways to the mechanosensory periphery causes an attenuation of stimulus encoding in vestibular and lateral line afferent pathways during self-motion.

## Discussion

During rhythmic locomotor activity, cranial mechanosensory ENs fire in a cyclic burst pattern that derives from an efference copy drive from the spinal central pattern generator. This predictive intrinsic signal informs the hair cell sensory periphery in both the inner ear and neuromasts of the lateral line system about the temporal structure of the ongoing locomotor command. Despite a variable influence of locomotor corollary discharge on individual vestibular and lateral line afferents, in both cases the evoked population response during coincident head/body motion and hydrodynamic stimulation is reduced, commensurate with an adaption of sensory encoding to the altered stimulus magnitudes that occur during locomotion.

Active movements such as locomotion generate reafferent sensory signals that interfere with the detection and interpretation of concurrent extrinsically induced passive motion^38^,^39^. However, intrinsic neural copies of the actual commands that produce locomotor movements offer a convenient substrate for neural computations that account for the expected sensory outcome of active self-motion^40^. In this way, locomotor efference copy^8^ or corollary discharge^7^ is highly suited to influence the processing of head/body motion signals at the vestibular/lateral line sensory periphery as well as within associated central circuitry^38^.

The functional impact of intrinsic corollary discharges is particularly well understood in the mormyrid fish electrosensory system, which is evolutionarily closely related to the vestibular and lateral line systems^41^. During electric organ activity of weakly electric fish, corollary discharges of the electromotor commands suppress reafferent stimulation at the first central relay station in the cerebellum-like electrosensory lobe^42^,^43^. Moreover, the correct interpretation of external electrosensory signals is not only impaired by self-generated electric fields but also by body motion due to locomotor or ventilatory activity^44^,^45^,^46^,^47^,^48^. However, in the absence of efferent innervation of electroreceptors and their associated afferent fibres at the sensory periphery^41^, the influence of motor corollary discharges occurs entirely centrally, where these intrinsic signals generate cancelling negative images of the sensory consequences of the fish's own movements in neurons of the electrosensory lobe^48^.

In contrast to the electrosensory system, the mechanosensory endorgans of both the vestibular and lateral line systems are richly innervated by EN populations, thereby offering an additional possibility to influence signal encoding at the first neuronal level. Indeed, efferent pathways to peripheral sensors constitute an essential component for informing these movement-detecting systems about the altered stimulus conditions during locomotion^11^. However, despite known morphological, physiological and pharmacological properties of vestibular nerve efferent fibres^12^,^15^,^27^, their direct electrical or sensory activation has yielded widely differing effects on vestibular afferent fibre activity in various species and under diverse experimental conditions^13^,^20^,^21^,^23^,^24^,^28^,^29^,^30^,^49^. Consequently, the functional role of vestibular efferent innervation has so far remained enigmatic. The results of the present study therefore place these earlier disparate observations into perspective by identifying a context-dependent role for vestibular ENs during the expression of an essential and definable natural behaviour. While our discovery of rhythmic locomotor-related signals occurring in vestibular nerve efferents is novel, the activation of lateral line efferent fibres during swimming in dogfish and *Xenopus* has been previously reported^17^,^19^. Our data also demonstrate for the first time that cranial mechanosensory efferent pathways reliably inform both the lateral line and vestibular sensory peripheries about ongoing locomotor activity by conveying parallel neural replicas of the spinal CPG output to lateral line neuromasts and inner ear endorgans.

Significantly, the distinct behavioural context in which these efferent pathways are engaged is inscribed in the information content of their corollary activation (Fig. 3). As found in a number of other systems, efference copies of motor behaviours with relatively predictable outcomes either adapt the sensory periphery to an altered stimulus condition or compensate for unwanted sensory consequences of the behaviour in question^10^,^39^,^50^,^51^,^52^. The corollary discharge signal conveyed by vestibular and lateral line mechanosensory efferents is therefore ideally suited to notify peripheral hair cell targets about the precise dynamics of ongoing locomotor activity. The neural origin of this internal signal within the first 10 cord segments complies with the large undulatory head movements that result exclusively from the alternating left/right contractions of rostral tail and trunk muscles during swimming, as found previously for the spinal source of locomotor efference copy-driven eye movements^51^. However, although the corollary activity of ENs consists of discrete locomotor-timed bursts, these phasic signals are also likely to be converted into a more persistent postsynaptic hair cell/afferent fibre response, as shown in toadfish upon electrical activation of its mechanosensory efferent system^13^,^20^,^21^.

**Figure 7:**
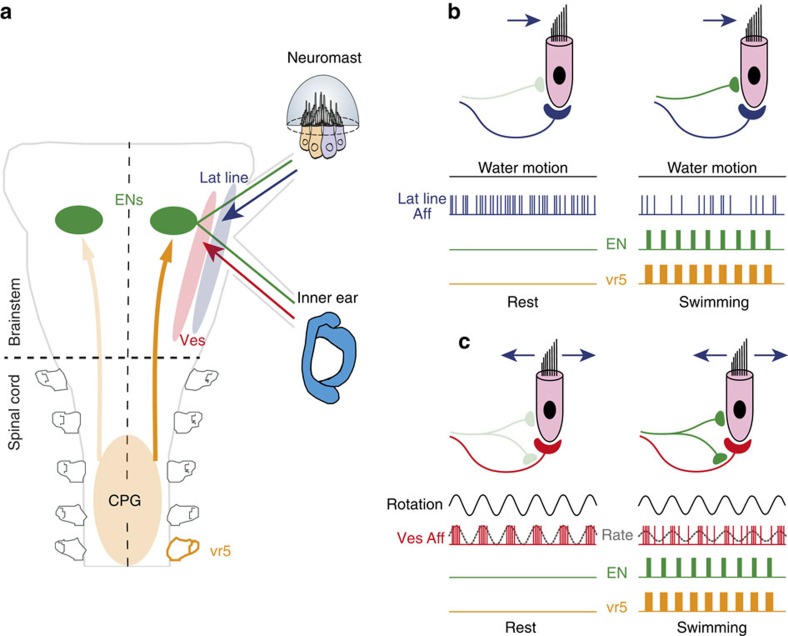
Schematics summarizing the functional impact of locomotor corollary discharge on lateral line and vestibular mechanosensory signalling. (**a**) Connectivity between spinal locomotor CPG circuitry, mechanosensory efferent neurons (ENs) and the sensory periphery along with afferent projections to central vestibular and lateral line nuclei. (**b**,**c**) Influence of locomotor activity conveyed by ENs to lateral line (**b**) and vestibular (**c**) hair cells/afferent innervation on stimulus signal encoding. The afferent responsiveness to either constant water motion (**b**—left) or cyclic head rotation (**c**—left) is substantially attenuated (**b**,**c**—right) during concomitant locomotor CPG activation.

The previously reported effects of experimentally elicited vestibular efferent discharge on afferent firing patterns^20^,^23^,^24^,^30^,^49^,^53^ comply with the variable influences of locomotor corollary EN activation found in the current study. However, despite the diverse effects of efferent firing on the spontaneous activity of vestibular and lateral line afferents, the overall mechanosensory responsiveness of both afferent populations is significantly attenuated during locomotor activity. The finding that the resting rates of vestibular afferents may either decrease, remain unaltered or even increase in response to EN firing is possibly related to the bilateral push–pull organization of the vestibular system, in contrast to the lateral line system. In the semicircular canal system, any imbalance in afferent signalling between the two sides is interpreted centrally as resulting from head rotation^5^. Thus, maintaining bilaterally symmetrical global rates of afferent fibre resting activity by averaging out the opposing effects of locomotor corollary efferent signals would in turn ensure equilibrated resting activity within the bilateral central vestibular circuitry, in accordance with an underlying principle for effectively encoding angular motion in space^4^,^5^. In contrast to vestibular (semicircular canal) sensory processing, which relies on bilateral organs for differential neural computations, the effective encoding of water motion in central lateral line nuclei only requires single patches of neuromasts containing hair cells with opposite polarities. The latter are innervated by separate lateral line afferent fibres, and thus comprise distinct perceptive entities that allow encoding bidirectional water motion without the necessity to extract integrative signals from bilateral comparisons^54^. The substrates for encoding head/body movement and water motion are also paralleled by differences in their respective efferent innervation patterns. While lateral line ENs connect uniquely with their hair cell targets, vestibular ENs make synaptic connections with both hair cells and the afferent pathways that serve them^55^ (Fig. 7). This latter dual innervation pattern coupled with an apparent greater pharmacological diversity in target influence^20^,^23^,^27^,^56^ again points to a potentially more variable functional outcome of efferent pathway activation for vestibular signal encoding.

Unlike the evident adaptive tuning of motion encoding in vestibular afferent fibres during locomotion in larval *Xenopus*, a corresponding efference copy influence on afferent discharge modulation during active head motion in primates has not been encountered^57^,^58^. While this difference might be related to species-specific diversity in neuronal computational requirements, it is more likely to be due to the difference in neural origins of the two underlying motor programmes. In *Xenopus*, rhythmic locomotor behaviour originates from a spinal CPG network whose associated corollary discharge is conveyed by ascending spino-cerebral pathways that are likely to be the same as those that drive compensatory eye movements^51^. In monkeys, however, voluntary head movements are driven by descending cortical commands^38^,^59^,^60^. Even though there is no difference between vestibular afferent encoding of active and passive head movements, motor efference copies together with proprioceptive inputs during voluntary neck movements, which likely originate from descending cortico-spinal pathways, cause a suppression of sensory inputs in primate central vestibular neurons^28^, thereby differentiating the two motion components. Thus, depending on the origin and nature of a motor programme for self-motion, an accompanying efference copy may exert its influence on reafferent sensory signalling at different, yet potentially overlapping levels of the nervous system. Since spinal CPG-derived efference copies were probably already present in aquatic vertebrate ancestors as evidenced by current protochordate lineages^61^,^62^, a corollary influence on mechanosensory afferent encoding via an associated efferent system is likely to represent an evolutionarily conserved condition that might also be effectively implemented during primate locomotion. Interestingly, supporting evidence for this idea comes from previous clinical studies in which human subjects with and without a vestibulopathy expressed a more stable posture during running than during walking^63^,^64^. This observation led to the conclusion that spinal locomotor signals might exert a direct influence on the vestibular sensory periphery, very reminiscent of the effects demonstrated in our study. Therefore, and in line with a parallel anecdotal report on a vestibular-impaired dog^63^, an adaptation of neural encoding at the sensory periphery during locomotion may serve as a general mechanism among vertebrates.

## Methods

### Experimental animals

Experiments were performed on semi-isolated *in vitro* preparations of larval *Xenopus laevis* at stages 48–55 (ref. 65) in compliance with the ‘Principles of Animal Care', publication by the National Institute of Health and the German law for animal protection (Tierschutzgesetz). Permission for the *in vitro* experiments was granted by the Regierung von Oberbayern (55.2-1-54-2531.3-18-10). All animals were obtained from the in-house breeding facility at the Biocenter Martinsried of the LMU Munich.

### Preparations

In all experiments, animals were first anaesthetized in 0.02% 3-aminobenzoic acid ethyl ester (MS-222; Sigma-Aldrich, Germany) in ice-cold frog Ringer (composition in mM: NaCl, 75; KCl, 25; CaCl_2_, 2; MgCl_2_, 0,5; NaHCO_3_, 25; glucose, 11; pH 7.4). The ventral part of the skull, including the jaw, was carefully removed with the tail remaining attached to the head. Preparations were transferred to a Sylgard-lined Petri dish and the skin covering the dorsal head surface was removed, the soft skull tissue opened and the forebrain disconnected. The rostral spinal cord and ventral roots until segment 20 were exposed, then roots 1–20 were disconnected from the tail/trunk musculature and the cord region was isolated from the surrounding tissue. In some preparations, the remaining caudal part of the tail was firmly secured with insect pins to the Sylgard floor at the level of segments 21–25 with the caudal part left free to perform undulatory swimming-related movements. Preparations were rinsed in fresh Ringer solution, transferred to a Sylgard-lined Petri dish (volume 5 ml) and continuously superfused with oxygenated Ringer solution at a rate of 1.3–2.1 ml min^−1^. The temperature of the bathing solution was maintained at 17±0.2 °C.

### Electrophysiology

*Fictive swimming*. Motor output of the spinal locomotor CPG in such semi-isolated preparations was monitored in spinal ventral roots (vrs) recorded uni- or bilaterally from cord segments 3–18 during episodes of so-called ‘fictive swimming', the neural correlate of actual behaviour that has been previously established in a number of animal model systems including locomotion in lamprey^66^ and *Xenopus*^34^,^67^,^68^ and vocalization in toadfish^69^. In addition to the mostly spontaneous expression of fictive swimming under such *in vitro* conditions, and to more predictably obtain swimming episodes, electrical stimulation of the head and caudal part of the tail was occasionally used to instigate locomotor sequences. Electrical stimuli were generated with an integrated stimulus isolation unit (STG 4004, Multichannel Systems, Germany) and consisted of trains of 2–10 pulses (0.2 ms, ∼100 μA at 100 Hz) that were delivered through a pair of Teflon-coated silver wires (diameter: 0.76 mm; AG 25-T, Science Products, Germany).

*Mechanosensory efferent activity*. To record vestibular (VIIIth cranial) nerve efferent activity, the otic capsule on one or both sides was opened and the anterior and/or posterior branches of the vestibular nerve (AVN and PVN) were carefully isolated from their respective endorgans and cleaned from surrounding tissue. To record lateral line efferent fibre activity, the anterior and posterior lateral line nerves (ALLNs and PLLNs) were exposed bilaterally outside the brain case and disconnected from the sensory periphery.

*Mechanosensory afferent activity*. The potential influence of locomotor-related efferent fibre activity on afferent mechanosensory encoding was assessed in semi-isolated preparations with still intact sensory organs (inner ear and lateral line) and hair cell afferent connectivity. *En passant* recordings from mechanosensory afferents and, in a few fortuitous cases, efferent nerve fibres were made during hydrodynamic or imposed head/body motion-driven sensory stimulation, while episodes of fictive locomotion were recorded conjointly from spinal ventral roots.

### Vestibular afferent activity

Recordings from semicircular canal afferent fibres were made in preparations with intact otic capsules and functional inner ear endorgans in the absence and presence of fictive swimming. Semi-isolated preparations were secured to the Sylgard floor of a recording chamber with the ventral side up. A ventral opening of the cranium gave access to the VIIIth nerve between the intact otic capsule and the hindbrain. This allowed *en passant* recordings of sensory stimulus-evoked activity of vestibular nerve afferent fibres to be made in a condition where the connectivity of efferent fibres and the sensory periphery were preserved. For application of rotational stimuli, the recording chamber was mounted on a computer-controlled, motorized two-axis turntable (Acutronic Deutschland GmbH). Motion stimuli consisted of sinusoidal rotations around the yaw and roll axis at frequencies of 0.5–1 Hz with corresponding peak velocities of ±30–60° s^−1^.

### Lateral line afferent activity

Recordings from the PLLN were made in preparations with intact tail musculature and cutaneous neuromasts at the dorso-lateral region of the head/tail. Transecting the spinal cord caudal to segment 15 prevented any potential residual motion artefacts during fictive swimming. For *en passant* recordings of afferent neuron activity, a short (∼0.5–1 mm) section of the PLLN branch was detached from the skin and cleaned from surrounding tissue. Nerve afferent activity was recorded in the absence and presence of hydrodynamic stimuli. For the latter, neuromast hair cells on the skin surface were stimulated by a constant Ringer flow (∼10 mm s^−1^) that was directed rostro-caudally along the surface of the preparation.

*Electrophysiological recordings*. All extracellular recordings, including *en passant* recordings of vestibular and lateral line afferent and efferent fibres, were made with glass suction electrodes fabricated with a horizontal puller (P-97 Brown/Flaming). To optimize recordings of spike discharges (both single- and multiunit) in spinal vrs (from segments 3 to 18) and the central stumps of mechanosensory nerve branches, the tip diameter of electrodes was individually adjusted to match the respective nerve size. For *en passant* recordings, electrodes were broken back to a tip size of ∼2 μm. Recorded activity was amplified (EXT 10-2F; npi electronics, Tamm, Germany), digitized at 10 kHz (CED 1401, Cambridge Electronic Design, Cambridge, UK), processed with commercial software (Spike 2, Cambridge Electronic Design), stored on a PC and analysed offline.

*Recording analysis*. Recordings were analysed using Igor pro software (Wavemetrics, USA) and custom-written macros. Spike time measurements were used to calculate the instantaneous frequency for spinal vr, vestibular (VIIIth cranial) and lateral line nerve activity. Rate measurements included all spikes in a given multiunit recording. The discharge of mechanosensory ENs was compared with the corresponding phase of the swimming cycle by triggering instantaneous frequency measurements from the onset of each associated vr burst. The timing of EN firing relative to vr activity was transformed into a phase angle and displayed as a polar plot in which the direction and length of an individual vector indicated the phase (0°, synchrony; 180°, alternation) and strength of coupling, respectively. Multiple episodes of locomotor activity were analysed for each nerve recording with at least 10 cycles of stable fictive swimming per episode. The total duration of efferent neuronal and vr activity was defined as the time between the first and last bursts of a given fictive swimming episode. The frequency of rhythmic vr and EN bursting was calculated from the inverse of the interval between consecutive bursts in each case. The relationship between the magnitudes of vr and EN discharge was determined by calculating the respective integral from the raw vr and mechanosensory nerve recordings using a bin width of 10 ms. Due to the variable number of monitored axons in the different vr recordings, integrals were normalized within each animal. The timing of burst integral peaks was also used to separate single-phase (1:1 matching between efferent and vr burst rhythms) from biphasic (two efferent bursts per vr burst cycle) coupling patterns.

### Lesion experiments

To identify the neural trajectories that convey corollary discharge signals from locomotor centres to the hindbrain mechanosensory efferent nuclei, various combinations of surgical lesions were made in semi-isolated central nervous system preparations. In a first set of experiments, the midbrain was removed by a transection of the brainstem rostral to the cerebellum, followed by a spinal hemisection immediately caudal to the obex. In a second series, successive complete transections of the spinal cord were made from vr20 in various step sizes until vr5. Following each surgical intervention, the preparation was allowed to recover for a period of 30 min before recording of neuronal activity commenced. After completion of physiological recordings, preparations were fixed in 4% paraformaldehyde in 0.1 M phosphate buffer (pH 7.4) for 5–6 h and preserved for *post hoc* verification of lesion specificity by whole-mount light microscopy.

### Central anatomy of mechanosensory ENs

The hindbrain segmental location and topographical organization of ENs with axonal projections in the different lateral line and vestibular nerve branches were determined by retrograde transport following application of fluorescent tracers (Alexa Fluor 488, 546 dextran, Life technologies, USA) in various combinations to the cut ends of the mechanosensory nerves in semi-isolated *in vitro* preparations^70^. Crystals of the tracers, melted onto the tip of an injection needle were inserted into the lateral line nerves close to the cranial exit of the ALLN and PLLN roots or into one of the two vestibular nerve branches (AVN or PVN) after opening of the otic capsule. Following incubation for 24–48 h in oxygenated Ringer solution at 14 °C, preparations were fixed in 4% paraformaldehyde in 0.1 M phosphate buffer (pH 7.4) at 10 °C for 5–6 h and rinsed (3 × 10 min) in cold 0.1-M PBS (PBS, pH 7.4). The brainstems were removed, cleaned of surrounding tissue, mounted on slides and coverslipped using Vectashield (Vector Laboratories, Burlingame, USA). The labelled somata and central projections of mechanosensory ENs and afferent axon terminals were reconstructed from stacks of optical sections obtained from scanning on a confocal microscope (Leica SP5). *Z* axis projections were generated using the ImageJ software package (http://fiji.sc/wiki/index.php/Fiji). To map the position of retrogradely labelled ENs onto the hindbrain segmental scaffold, preparations were scanned with an illumination wavelength of 612 nm to demark rhombomere outlines.

### Ca^2+^ imaging of mechanosensory ENs

EN cell bodies were retrogradely loaded with Calcium Green-1 dextran (Invitrogen, Eugene, OR, USA) applied as crystals to the peripheral ending of the AVN 24 h prior to an experiment. Imaging of Ca^2+^ transients was performed with an epifluorescence microscope (Axio Examiner Z1, Carl Zeiss, Germany) and a CCD camera (Axiocam Hsm, Carl Zeiss) in both the absence and presence of locomotor activity. To prevent potential movement artefacts during imaging, all residual muscular elements of preparations were removed. Images were captured at a rate of 10–20 frames s^−1^ (Axiovision, Zeiss), stored and analysed *post hoc* using the MBF-ImageJ Java software package (http://rsb.info.nih.gov/ij/) and custom-written scripts. The background fluorescence was subtracted, and bleaching effects were corrected using a linear regression algorithm. All data were presented as relative changes in fluorescence (Δ*F*/*F*). The duration of an individual Ca^2+^ transient was taken as the time at half-maximal amplitude of the fluorescence change during a given swimming episode.

## Additional information

**How to cite this article:** Chagnaud, B. P. *et al*. Spinal corollary discharge modulates motion sensing during vertebrate locomotion. *Nat. Commun.* 6:7982 doi: 10.1038/ncomms8982 (2015).

## Supplementary Material

Supplementary Information Figures 1-3.
