# Supplementary material for: Spinal corollary discharge modulates motion sensing during vertebrate locomotion
Source: Nat Commun. 2015 Sep 4;6:7982. doi: 10.1038/ncomms8982 (PMC4569702; doi:10.1038/ncomms8982)
Supplement: Supplementary Information — Figures 1-3. [file ncomms8982-s1.pdf]

Supplementary figure 1

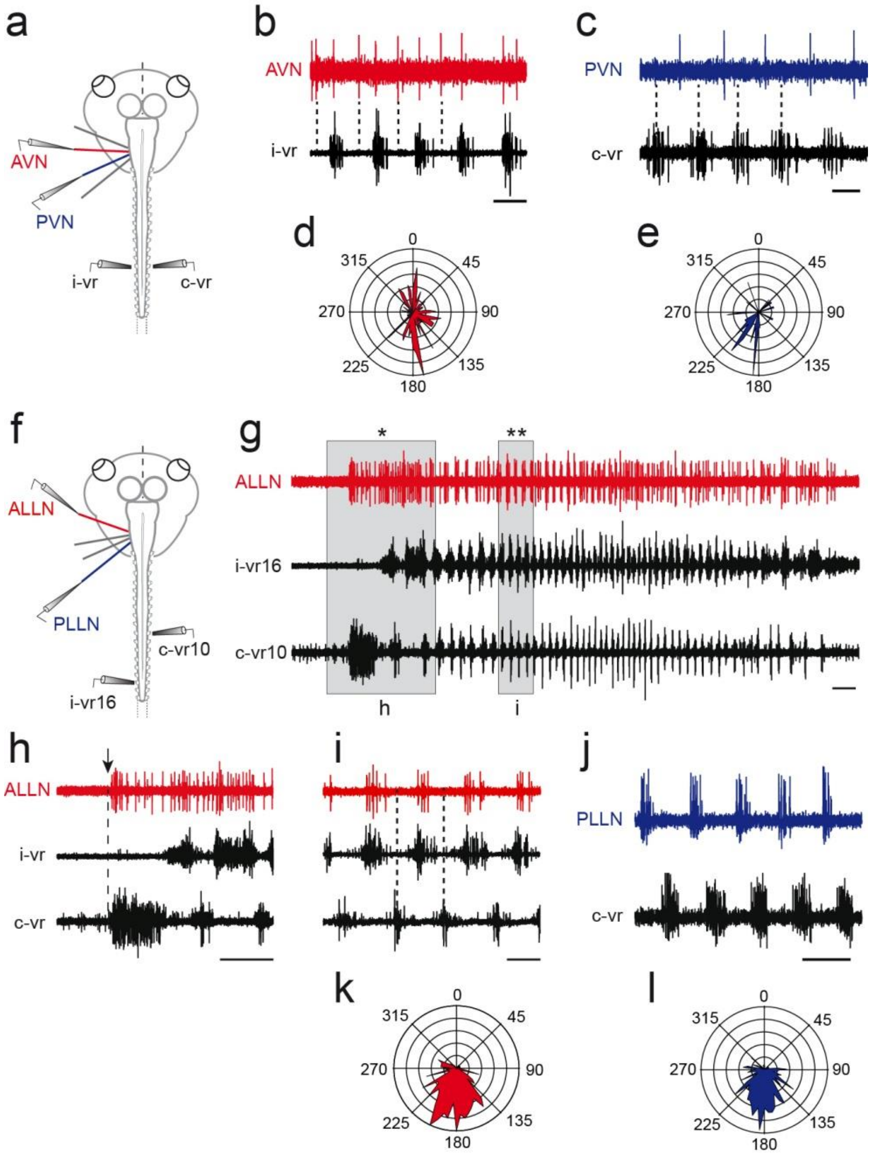

Supplementary figure 1. Coupling of spinal vr and mechanosensory nerve discharge during fictive locomotion in semi-isolated *Xenopus* tadpole preparations (a,f). b,c,g-j, Simultaneous recordings of various combinations of a left/right spinal vr and the left AVN (b), PVN (c), ALLN (g-i) or PLLN (j) showing activity coordinated mainly in phase opposition (see corresponding polar plots in d,e,k,l). Note that a bi-phasic (coincident as well as alternating) coupling pattern was occasionally observed (dashed line in b; polar plot in d). The initial discharge at episode onset (*) and subsequent expression of regular rhythmic bursting (**; grey areas in g) are shown

for simultaneously recorded ALLN and bilateral vrs on an extended time scale in h and i, respectively. After initial tonic firing at swim episode onset (h), the ALLN (i) and PLLN (j) express bursting that remains in strict phase opposition with bursts in the c-vr (see black dashed lines in l and corresponding plots in k and l).

Supplementary figure 2

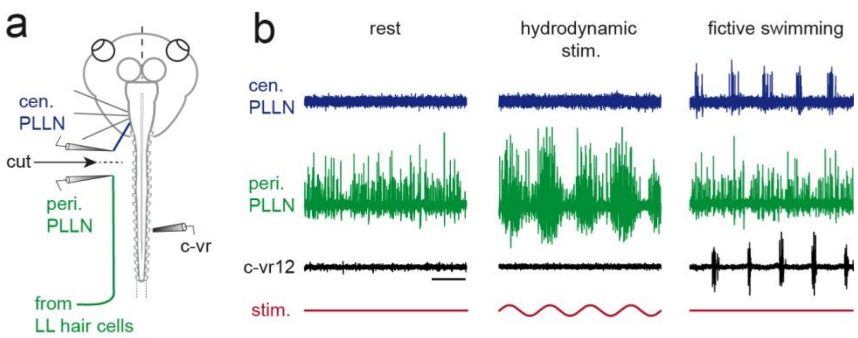

Supplementary figure 2. Identification of efferent and afferent mechanosensory activity in a semi-isolated *in vitro* preparation (a). b, Simultaneous recordings of a spinal vr (c-vr12; black trace in b) and the central (blue in a,b) and peripheral PLLN stumps (green in a,b) after transection of the nerve outside the cranium in a semi-isolated preparation with still intact neuromasts. Recordings in b were made at rest (left panel), during sinusoidal hydrodynamic stimulation (lower red trace) of the neuromasts (middle panel), and during fictive locomotion (right panel). Calibration bar in b: 0.2 s.

Supplementary figure 3

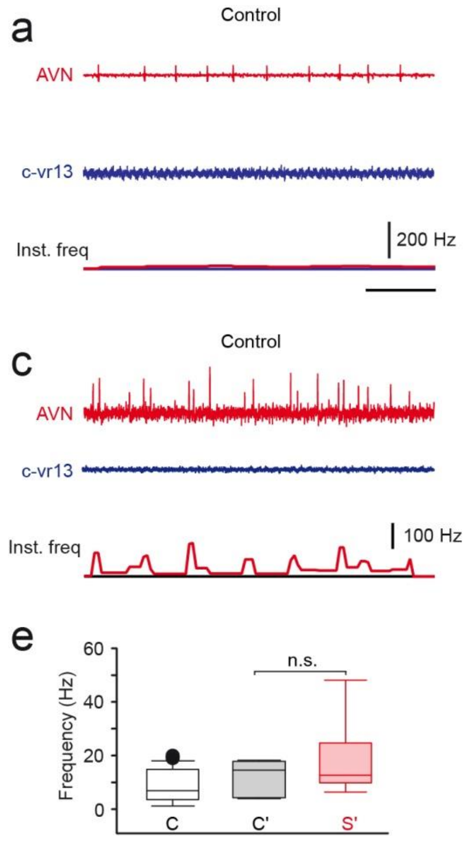

Supplementary figure 3. Vestibular afferent neuron firing at rest and during fictive swimming. a-d, Spontaneous afferent discharge (red traces; upper, raw; lower, instantaneous frequency) in the AVN of controls (a,c) either increases (b) or decreases (d) during locomotor-related rhythmic bursting in a contralateral spinal vr (blue traces). e,f, Box and whisker plots comparing resting rates (e) and discharge regularity (cv^2; f) of all recorded afferent fibers (C; n = 22) in the absence of fictive swimming, and of a subpopulation (n = 6) that was successfully recorded both before (C') and during fictive swimming (S'). Horizontal calibration bar: 0.2 s for all traces.
